# Multinational proficiency tests for *EGFR* exon 20 insertions reveal that the assay design matters

**DOI:** 10.1038/s41598-024-63821-2

**Published:** 2024-06-06

**Authors:** Michaela A. Ihle, Carina Heydt, Anne Maria Schultheis, Robert Stöhr, Florian Haller, Sylvia Herold, Daniela Aust, Wolfgang Dietmaier, Matthias Evert, Markus Eszlinger, Anja Haak, Silke Laßmann, Daniela Vorholt, Frank Breitenbücher, Martin Werner, Anna Streubel, Thomas Mairinger, Maja Grassow-Narlik, Sabine Merkelbach-Bruse

**Affiliations:** 1 Institute of Pathology, University of Cologne, Faculty of Medicine and University Hospital Cologne, Kerpener Str. 62, 50924 Cologne, Germany; 2 Institute of Pathology, University Hospital Erlangen, Krankenhausstr. 8–10, 91054 Erlangen, Germany; 3 Institute of Pathology, Hospital of the Technical University Dresden, Fetscherstr. 74, 01307 Dresden, Germany; 4 Institute of Pathology, University of Regensburg, Franz-Josef-Strauss-Allee 11, 93053 Regensburg, Germany; 5 Institute of Pathology, University Hospital Halle, Magdeburger Str. 14, 06112 Halle (Saale), Germany; 6 Institute of Surgical Pathology, Medical Center Freiburg, Breisacherstr. 115a, 79106 Freiburg, Germany; 7 Janssen-Cilag GmbH, Johnson&Johnson Platz 1, 41470 Neuss, Germany; 8 Institute of Tissue Diagnostics, MVZ at Helios Klinikum Emil Von Behring, Walterhöferstr. 11, 14165 Berlin, Germany; 9 Quality Assurance Initiative Pathology (Qualitätssicherungs-Initiative Pathologie [QuIP®]), Reinhardtstr. 1, 10117 Berlin, Germany

**Keywords:** Molecular medicine, Lung cancer

## Abstract

Insertion mutations in exon 20 of the epidermal growth factor receptor gene (*EGFR* exon20ins) are rare, heterogeneous alterations observed in non-small cell lung cancer (NSCLC). With a few exceptions, they are associated with primary resistance to established EGFR tyrosine kinase inhibitors (TKIs). As patients carrying *EGFR* exon20ins may be eligible for treatment with novel therapeutics—the bispecific antibody amivantamab, the TKI mobocertinib, or potential future innovations—they need to be identified reliably in clinical practice for which quality-based routine genetic testing is crucial. Spearheaded by the German Quality Assurance Initiative Pathology two international proficiency tests were run, assessing the performance of 104 participating institutes detecting *EGFR* exon20ins in tissue and/or plasma samples. *EGFR* exon20ins were most reliably identified using next-generation sequencing (NGS). Interestingly, success rates of institutes using commercially available mutation-/allele-specific quantitative (q)PCR were below 30% for tissue samples and 0% for plasma samples. Most of these mutation-/allele-specific (q)PCR assays are not designed to detect the whole spectrum of *EGFR* exon20ins mutations leading to false negative results. These data suggest that NGS is a suitable method to detect *EGFR* exon20ins in various types of patient samples and is superior to the detection spectrum of commercially available assays.

## Introduction

*EGFR* exon20ins are rare somatic alterations observed in NSCLC, which make up between 2 and 12% of all *EGFR* gene alterations^1–3^. They are a largely heterogeneous group of mutations and most frequently occur in the region encoding for amino acids Asp761 to Cys775 of the C-helix domain and the loop following the C-helix domain^1,4,5^. With a few exceptions, *EGFR* exon20ins are generally associated with resistance to first-, second- and third-generation EGFR TKIs^1^. A key challenge in the detection of patients with *EGFR* exon20ins mutation is the large heterogeneity of these mutations. First-generation EGFR inhibitors (gefitinib and erlotinib) turned out to be an ineffective therapeutic option in the vast majority of patients harboring an *EGFR* exon20ins mutation^3^. Only few exceptions, e.g. the p.Ala763_Tyr764insPheGlnGluAla mutation, seem to respond to these inhibitors whereas the most common *EGFR* exon20ins mutations e.g. p.Ala767_Val769dup, p.Ser768_Asp770dup and p.Asn771_His773dup show high IC50 values (IC50 > 100) for erlotinib and gefitinib^6^. For second-line inhibitors (e.g. neratinib, dacomitinib and afatinib) there is limited clinical evidence for the effective use in patients with *EGFR* exon20ins mutations. Regarding third-generation EGFR inhibitors, Poziotinib is one of the most advanced inhibitors showing response rates against e.g. p.Asp770insAsnProGly or p.Asn771delinsPheHis^6^. Osimertinib shows high IC50 values (IC50 > 100) for the p.Ser768_Asp770dup and p.Asn_771_His773dup mutations and intermediate IC50 values (IC50 < 10 < 100) for e.g. p.Ala_Val769dup. However, these third-generation EGFR inhibitors also demonstrated activity against wildtype EGFR which may cause dose limitations in patients with *EGFR* exon20ins mutations.

Fortunately efficacious targeted therapies are slowly emerging: in December 2021, the bispecific antibody amivantamab was the first of its kind to be approved by the European Commission for the treatment of patients with advanced NSCLC and activating *EGFR* exon20ins, after failure of platinum-based therapy^7^. While the TKI mobocertinib is available for the same indication in the United States (US) as well as the United Kingdom (UK), the European marketing authorization application was withdrawn by the manufacturer in July 2022^8,9^. Further therapeutic approaches targeting *EGFR* exon20ins are currently in the pipeline^10^.

To identify patients eligible for treatment with these innovative targeted therapies, molecular testing is a prerequisite. The three most common *EGFR* ex20ins mutations account for less than 50% showing the high heterogeneity of this group of mutations. Therefore, reliable as well as sensitive detection methods are needed. Methodological standardization is of utmost importance to ensure the accuracy and reproducibility of molecular testing, especially when performed in a decentralized manner. Activities to achieve this goal include internal quality assessments, e.g., routine testing of known positive and negative samples, as well as external quality assessments, e.g., regular participation in proficiency testing.

Since patient data are often analyzed according to the type of mutation—for example, treatment responses may differ depending on the alteration present—, *EGFR* exon20ins, as other mutations, must be described in a standardized manner. To enable the consistent identification of patients carrying the same aberrations, the Human Genome Variation Society (HGVS) proposed guidelines for the unambiguous description of sequence variants^11,12^. In this context, a duplication has to be prioritized when a variant can be described as either duplication or insertion, and the most possible 3′ position should be used for all descriptions of variants^11,12^.

QuIP®, a joint venture of the German Society of Pathology (Deutsche Gesellschaft für Pathologie, DGP) and the Federal Association of German Pathologists (Bundesverband Deutscher Pathologen, BDP), coordinates regular proficiency tests to monitor performance quality of institutes in the field of diagnostic immunohistochemistry and molecular pathology. Here, we report the results of two such proficiency tests, assessing the performance of a total of 104 international pathology institutes with regard to the detection of a pre-specified set of *EGFR* exon20ins variants present in either formalin-fixed, paraffin-embedded (FFPE) tissue or liquid biopsy samples.

## Results

### Selection of reference samples

**Figure 1:**
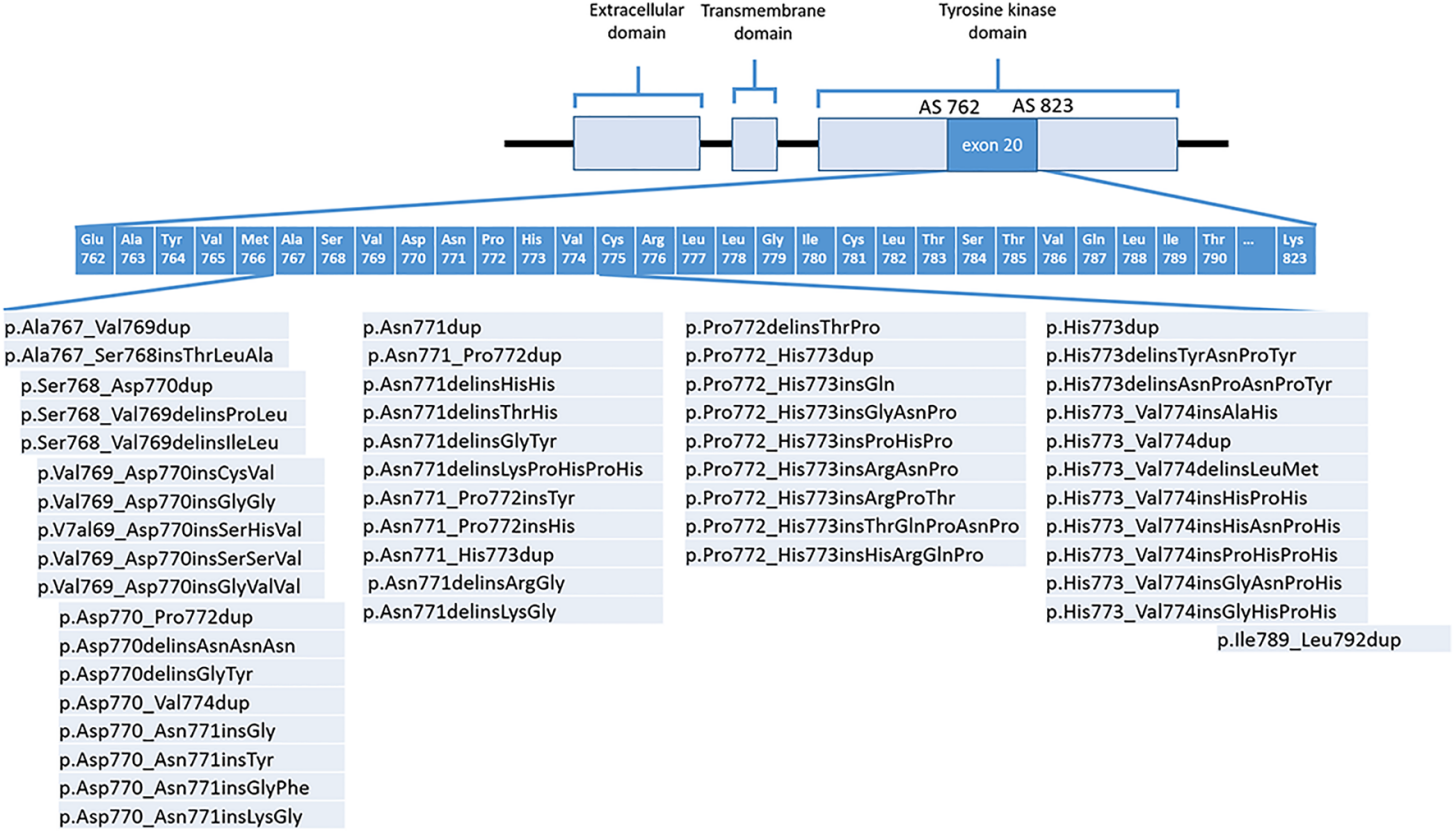
Spectrum of *EGFR* exon20ins. Insertions in exon 20 of the *EGFR* gene are heterogeneous: in a patient cohort from Cologne (n = 203; recorded at the Institute of Pathology of the University of Cologne, Faculty of Medicine, and University Hospital Cologne), a total of 50 distinct *EGFR* exon20ins variants were identified on the protein level, covering the entire C-helix as well as the loop following the C-helix. Proficiency test samples were chosen based on these findings to feature a wide range of different *EGFR* exon20ins variants occurring in clinical practice. Ref-Seq ID: NM_005228.

**Figure 2:**
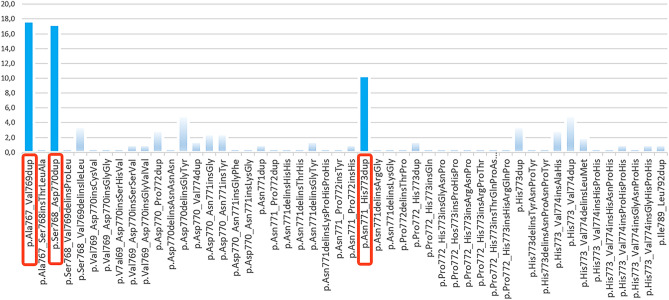
Prevalence of *EGFR* exon20ins**.** In a patient cohort from Cologne (n = 203; recorded at the Institute of Pathology of the University of Cologne, Faculty of Medicine, and University Hospital Cologne), a total of 50 distinct *EGFR* exon20ins variants were observed on the protein level, with prevalence ranging between ~ 0.5% and ~ 5% of all *EGFR* exon20ins (excluding the three most common alterations). The most common *EGFR* exon20ins occurring in this cohort were c.2300_2308dup (p.Ala767_Val769dup; 17.7%), c.2303_2311dup (p.Ser768_Asp770dup; 17.2%), and c.2311_2319 dup (p.Asn771_His773dup; 10.3%). Approximately 50% of the detected *EGFR* exon20ins have a prevalence of < 1%. Ref-Seq ID: NM_005228.

Reference samples for the external proficiency tests were chosen based on the mutational spectrum of *EGFR* exon20ins and the respective prevalence detected in a patient cohort from Cologne upon performing a retrospective database analysis (Figs. 1 and 2; Table S1). Overall, 203 patients with a total of 56 different *EGFR* exon20ins were identified; prevalence ranged between 0.5 and 17.7% within this group of patients. The mutations with the highest prevalence were chosen for the proficiency tests as they are clinically most relevant and cover the heterogeneity of *EGFR* exon20ins mutations.

To simulate real-life conditions, i.e., to ensure that participating institutes were able to detect low allelic fractions potentially occurring in patients´ samples, different allelic fractions were chosen for the liquid biopsy samples. According to the German uniform assessment standard (Einheitlicher Bewertungsmaßstab, EBM) regulating the reimbursement of services provided by statutory health insurance-(SHI)-accredited physicians, the *EGFR* exon20ins detection method of choice must have a limit of detection (LOD) ≤ 1%^13^. The minimal allelic fraction and the region to be covered by this proficiency test was stated in the instructions. Each participant could check whether the established test would be suitable for this proficiency test. The responsibility for choosing a suitable assay covering the mutational spectrum and the limit of detection requested rested with the respective participants.

### Assessment of laboratory performance (DACH proficiency test)

A total of 37 institutes signed up for participation in the DACH proficiency test. After receiving their respective test sets, 19 institutes submitted results for both methodological parts, tissue and liquid biopsy, while 18 institutes submitted results for the tissue part only.

#### Tissue part

**Figure 3:**
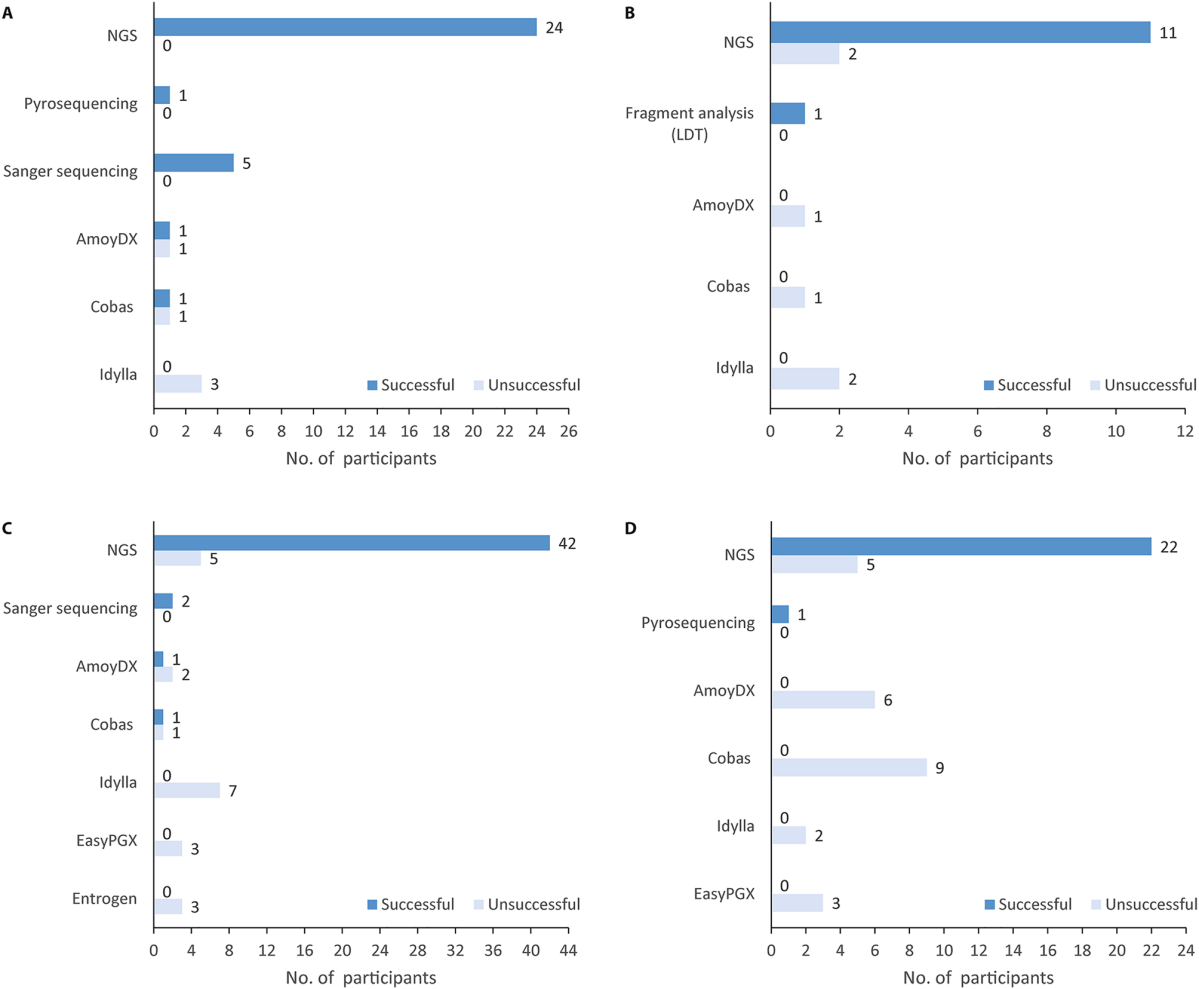
Overview of proficiency test outcomes depending on methods used for *EGFR* exon20ins testing. Successful participation in the proficiency tests did not depend on the kit used to extract (cf)DNA, but on the method subsequently used to detect *EGFR* exon20ins. Overall, institutes utilizing NGS and Sanger sequencing had higher success rates than institutes employing mutation-/allele-specific (q)PCR. Panel (**A**) shows the results of the tissue part of the DACH proficiency test. All participants using NGS, pyro- or Sanger sequencing as method of choice passed, whereas only 2 of the 7 institutes using mutation-/allele-specific (q)PCR did. Panel (**B**) displays the outcomes of the liquid biopsy part of the DACH proficiency test. None of the participating institutes using mutation-/allele-specific (q)PCR passed, while 84% of the institutes utilizing NGS participated successfully. Panel (**C**) shows the results of the tissue part of the international proficiency test: 89.8% of the participating institutes employing NGS or Sanger sequencing as their method of choice passed the quality control test, whereas only 2 of the 18 institutes (11.1%) using mutation-/allele-specific (q)PCR did. Panel (**D**) displays the outcomes of the liquid biopsy part of the international proficiency test. None of the participating institutes utilizing mutation-/allele-specific (q)PCR or pyrosequencing passed, while 81.5% of the institutes using NGS participated successfully. Details on suppliers can be found in the Supplement.

Out of the 37 institutes submitting results, 32 (86.5%) received certificates of successful participation. Samples 1, 4 and 5 were most often falsely described as wildtype when in fact carrying *EGFR* exon20ins (Table S2). Successful participation did not depend on the DNA extraction kit used by participants, but rather on the subsequently employed *EGFR* exon20ins detection method (Fig. 3A). NGS (65%) and mutation-/allele-specific quantitative (q)PCR methods (19%) were used more frequently than Sanger sequencing (13%) or pyrosequencing (3%). All institutes using NGS, pyro- or Sanger sequencing passed the proficiency test, while only 2 of the 7 institutes (28.5%) using commercially available mutation-/allele-specific (q)PCR did (Table S3). Notably, all institutes misclassifying cases 1, 4 and 5 had employed PCR-based methods. No specific kit or platform was associated with successful participation: institutes utilized numerous devices and platforms as well as various kits or panels for the detection of *EGFR* exon20ins (Table S3).

Despite not influencing the performance rating, it is worth mentioning that sample 9 was incorrectly annotated by most participating institutes. In this sample, nucleotides 2300 to 2308 are duplicated, resulting in a duplication of amino acids 767 to 769 (p.Ala767_Val769dup). Yet, it was often classified as insertion instead of a duplication (Fig. S1). Furthermore, participants did not follow the HGVS 3′ rule according to which the most 3′ position possible of the reference sequence is arbitrarily assigned to be changed^11,12^. In this particular case, annotation leads to a shift of the mutation description by three base pairs towards the 3′ end of the gene.

The remainder of the samples were correctly annotated by most of the participating institutes.

#### Liquid biopsy part

Out of 19 participating institutes submitting results, 12 (66.7%) received certificates of successful participation. Samples 1, 2, 4, 5, 6, and 7 were most frequently misclassified as wildtype when in fact carrying *EGFR* exon20ins (Table S4). Case 2 had a low allelic fraction of 1%; eight institutes did not detect this mutation. Different commercially available kits were used to extract cell-free DNA (cfDNA) from plasma samples. Successful participation did not seem to depend on the extraction kit used but rather on the detection method subsequently employed (Fig. 3B). *EGFR* exon20ins detection methods included NGS (72%), fragment analysis (6%) and commercially available mutation-/allele-specific (q)PCR (22%). Multiple devices and platforms as well as kits or panels were used for the detection of *EGFR* exon20ins (Table S5). None of the institutes using commercially available mutation-/allele-specific (q)PCR passed the proficiency test, while 84% of the institutes utilizing NGS participated successfully (Table S5). One institute using fragment analysis (laboratory-developed test, LDT) passed.

### Assessment of laboratory performance (international proficiency test)

Of the 76 international institutes, which had registered to participate, 48 submitted results for both parts, 19 submitted results for the tissue part only, 1 submitted results for the liquid biopsy part only, and 8 did not submit any results at all. One institute submitted an empty questionnaire which counts as a submission; however, this institute was not considered in the subsequent analysis.

#### Tissue part

Of the 67 institutes entering test results for the tissue part, 46 (68.7%) received certificates of successful participation. Cases 4 and 5 were most often mislabeled as wildtype when in fact carrying *EGFR* exon20ins (Table S6). In line with the DACH proficiency test, participating institutes used different DNA extraction kits as well as different *EGFR* exon20ins detection methods (NGS: 70%, mutation-/allele-specific (q)PCR: 27%, and Sanger sequencing: 3%). Most institutes (89.8%) using NGS or Sanger sequencing passed the proficiency test, while only 2 of the 18 institutes (11.1%) using commercially available mutation-/allele-specific (q)PCR participated successfully (Fig. 3C). Institutes successfully using NGS or Sanger sequencing, passed regardless of the kit or platform that was chosen to detect *EGFR* exon20ins mutations (Table S7).

#### Liquid biopsy part

Of the 48 institutes submitting results for the liquid biopsy part, 22 (45.8%) participated successfully. False variant description occurred for all cases but one (case 3 [wildtype]; Table S6). None of the cases were removed from the current analysis, as mislabeling of the samples was not a consequence of material-related problems, but rather a problem of applying an inadequate detection method.

Various commercially available kits were utilized for cfDNA extraction; again, successful participation did not depend on the type of extraction kit used, but on the detection method employed afterwards (Fig. 3D). Molecular methods for *EGFR* exon20ins testing comprised NGS (56%), pyrosequencing (2%), and commercially available mutation-/allele-specific (q)PCR (42%)—different devices and platforms as well as kits and panels were used. None of the participating institutes utilizing either mutation-/allele-specific (q)PCR or pyrosequencing passed the proficiency test, while 22 of the 27 institutes (81.5%) using NGS participated successfully (Table S8). Although case 5 (p.His773dup) had a low allelic fraction of 2%, it was identified correctly by 87.5% of the participating institutes. Notably, 5 institutes using the Ion GeneStudio S5 as their NGS platform failed. However, this seems not to be a general problem with this platform as seven other institutes using the same platform succeeded in the proficiency test.

## Discussion

In clinical patient care setting, the detection of heterogeneous *EGFR* ex20ins mutations is challenging for molecular diagnostics laboratories. Two proficiency tests were performed to compare the results of *EGFR* ex20ins mutation detection of molecular diagnostic laboratories. Clinical NSCLC tissue and artificial liquid biopsy samples with and without *EGFR* ex20ins mutations were used: one test covered institutes from Germany, Austria, and Switzerland only (further referred to as “DACH”) and one covered institutes from Austria, Belgium, Estonia, France, Germany, Greece, Hungary, Ireland, Italy, Lithuania, Netherlands, Poland, Portugal, Russia, Slovakia, Switzerland, and Turkey (further referred to as “international”).

Success rate for the DACH proficiency test for the tissue part was almost 90% showing a high-quality standard in these countries. Most frequently NGS (65%) was used followed by mutation-/allele-specific (q)PCR (19%). In the international proficiency test success rate was lower with almost 70%. Again, most frequently used method of choice was NGS (70%) and mutation-/allele-specific (q)PCR (27%). However, 30% of the participants failed to succeed in the international proficiency test for the detection of *EGFR* ex20ins mutations indicationg the need to optimize molecular diagnostics. Appropriate and regularly updated validation, standardization and quality control guidelines might help achieve better results in the future^14^.

The results of the proficiency tests on liquid biopsy samples further confirms this observation. In the DACH proficiency test for liquid biopsy samples almost 70% of the participants succeeded, whereas in the international proficiency test only half of the participants succeeded. In both tests, method of choice was NGS again followed by mutation-/allele-specific (q)PCR. However, in the international proficiency test qPCR was used in 42% of participants; in the DACH proficiency test only 22% used qPCR.

Due to the wide range of different mutations as well as the overall rarity of *EGFR* exon20ins, the respective detection method necessitates sufficient analytical sensitivity, as well as specificity and needs to span the whole *EGFR* exon 20 region. Most commercially available mutation-/allele-specific (q)PCR kits, however, only detect a limited number of distinct *EGFR* exon20ins (Fig. S2); therefore, the spectrum of mutations covered by a specific test kit must be carefully scrutinized. NGS, in contrast, can cover the entirety of exon 20 of the *EGFR* gene with sufficient sensitivity to detect even low allelic fractions within the range of 1%.

In a sample cohort from Cologne, 54 different mutations were detected on DNA level, spanning nucleotides 2300 to 2378 and resulting in 50 distinct alterations on protein level (Fig. 2; Table S1). The three most common *EGFR* ex20ins mutations account for less than 50% showing the high heterogeneity of this group of mutations. This is in accordance with other publications: for example, Bauml et al.^1^ identified 40 unique *EGFR* exon20ins variants in a cohort of 175 patients^15^. In addition, Vyse and Huang as well as Yasuda et al.^16^ demonstrated that *EGFR* exon20ins occur throughout the entire region encoding the C-helix as well as the loop following the C-helix. In the cohort from Cologne, the mutations p.Ala767_Val769dup (17.7% of all *EGFR* exon20ins recorded), p.Ser768_Asp770dup (17.2% of all *EGFR* exon20ins recorded) and p.Asn771_His773dup (10.3% of all *EGFR* exon20ins recorded) occurred most frequently (Fig. 2; Table S1). Vyse et al.^1^ published similar numbers, and while Leal et al.^17^ described lower frequencies, they showed that p.Ala767_Val769dup and p.Ser768_Asp770dup were among the most frequent *EGFR* exon20ins. Yet, only p.Ala767_Val769 can be detected by all six commercially available mutation-/allele-specific (q)PCR kits shown in Fig. S2. In contrast, p.Ser768_Asp770dup can be detected by 5 of the 6 mutation-/allele-specific (q)PCR kits displayed and p.Asn771_His773dup is only covered by one of these assays. In line with this, 71% of the DACH region institutes and 88.8% of the international institutes using commercially available mutation-/allele-specific (q)PCR for the tissue parts failed their proficiency tests. Unsuccessful participation was not associated with a single system but could be observed across different PCR kits from various providers showing that the detection spectrum of these assays is not sufficient. In contrast, all DACH region institutes and 89.8% of the international institutes using NGS or Sanger sequencing for the tissue parts succeeded.

None of the institutes employing commercially available mutation-/allele-specific (q)PCR for their respective liquid biopsy parts passed. In contrast, the sole institute performing fragment analysis for the liquid biopsy part succeeded. Our data indicate that fragment analysis, in general, works for the detection of insertion mutations. However, it needs subsequent analysis to determine the exact underlying mutation as the different *EGFR* exon20ins mutations show different response rates to inhibitors^6,18,19^.

Confirming our observations, a recent publication demonstrated that PCR-based assays only detect about 50% of the *EGFR* exon20ins variants identified by NGS^15^. Similarly, Arcila et al.^20^ observed that the Idylla™ EGFR assay, while suitable for the detection of common *EGFR* mutations, did not detect certain *EGFR* exon20ins mutations identified by NGS. Most commercially available mutation-/allele-specific (q)PCR assays are simply not designed to detect the whole range of relevant *EGFR* exon20ins. If the probe-based mutation-/allele-specific (q)PCR lacks a probe for a specific mutation, the assay is not able to detect this mutation (Fig. S3). In a worst case scenario, up to 50% of patients with an *EGFR* ex20ins mutation would obtain false negative test results, consequently not receiving targeted treatment. Amivantamab, a bispecific antibody targeting MET and EGFR showed in a post platinum population a response rate of 40% and a progression free survival of 8.3 month. However, this targeted therapy is only approved for the treatment of patients with advanced NSCLC with activating *EGFR* ex20ins, after failure of platinum-based therapy. Therefore, the correct identification of *EGFR* ex20ins mutations is essential for therapeutic decisions.

Furthermore, results for reference sample 5 of the liquid biopsy part of the international proficiency test revealed that the limit of detection (LOD) might differ depending on the detection method used: as shown in Fig. S2, this specific mutation is covered by all six mutation-/allele-specific (q)PCR kits; however, none of the institutes using commercially available mutation-/allele-specific (q)PCR were able to identify it correctly. As it was stated in the proficiency test instructions that the minimal allelic fraction might be as low as 1%, institutes would have been required to choose a test that guarantees a sufficient limit of detection.

Interestingly, 2 of the 13 institutes using NGS in the DACH proficiency test failed to detect the mutation at an allelic fraction of 1% (case 2). As other participants with the same NGS platform or bioinformatics pipeline could detect these variants with low allelic fraction, This seems to be not a general NGS platform or pipeline specific problem. Furthermore, in the liquid biopsy part of the international proficiency test 5 institutes using the Ion GeneStudio S5 failed. However, seven other participants using the same platform succeeded in the proficiency test. It is unknown whether the variant was not called by the bioinformatic software tool, or if the variant was not detectable in the raw data.

The false-negative results may be caused by problems during cfDNA extraction, library preparation, sequencing or the bioinformatic analysis. To draw a final conclusion, more participants are needed with the same complete workflow to compare the whole analysis process. Our results indicate that both, the design of the method applied must be considered, and the entire analysis process needs to be validated under high quality standards to ensure sufficient sensitivity and specificity.

It is noteworthy to mention that in Europe, laboratories are generally free to choose the diagnostic method they use for the detection of molecular alterations (although recommendations have been issued for certain indications and biomarkers^21^). In contrast, the US FDA approves companion diagnostics together with the corresponding therapeutic product, or a non-companion diagnostic assay can be used in a laboratory that follows the Clinical Laboratory Improvement Amendments (CLIA), thereby restricting the freedom of methodological choice^22^. In case of *PIK3CA* mutation analysis in advanced breast cancer this approach has been shown to be possibly detrimental: in a study by Martinez-Saez et al.^23^, up to 95% of the patients potentially eligible for treatment with a PI3K inhibitor were missed by the mutation-/allele-specific (q)PCR kit approved as a companion diagnostic by the FDA. In comparison, two NGS-based tests detecting *EGFR* exon20ins are currently approved as companion diagnostics for targeted therapies, both of which have been shown to reliably identify patients benefiting from treatment with amivantamab^22,24^.

To consistently identify all patients with NSCLC potentially eligible for treatment with novel targeted therapies in clinical practice, the European Society for Medical Oncology (ESMO) recommends routine molecular diagnostic testing of tumor or plasma samples, using comprehensive NGS panels^21,25^, highlighting the priority of NGS in clinical routine practice. However, it seems that using NGS is not the current state-of-the-art in many laboratories. One reason might be high costs, the workload to establish this method and the high amount of bioinformatic data that need to be analyzed. Compared to NGS, mutation-/allele-specific (q)PCR and Sanger sequencing are easier to establish, have a shorter turn-around time and are accompanied by lower costs (Table 1).

**Table 1:** Comparison of methods used for *EGFR* ex20ins mutational analysis.

	Costs	Material requirements	Detection spectrum	Turn-around time	Data evaluation	Sensitivity	Specificity	Ease of implementation
NGS	+++	+++	+++	+++	+++	+++	+++	+
qPCR	++	+	+	+	+	++	++	++
Fragment length analysis	++	+	+	+	+	++	+	++
Sanger sequencing	+	+	+++	++	+	+	+++	+++

NGS, next generation sequencing; +++, high; ++, intermediate; +, low.

While correct labeling of the different variants according to HGVS guidelines was not related to performance in the proficiency tests, it is noteworthy to mention that participating institutes mainly disregarded the following three rules when naming *EGFR* exon20ins: (1) insertion of nucleotide repeats should be designated a duplication, (2) for all descriptions of variants, the most 3′ position possible should be used, (3) three letter code is preferred over one letter code^11^. For example, most participants did not use the correct nomenclature to describe the mutation in case nine of the national proficiency test (Fig. S1). There were more than six different descriptions of this particular mutation (correctly named p.Ala767_Val769dup). Whether this was due to bioinformatic errors or to human errors was not evaluated. However, some bioinformatic tools are known to be prone to incorrect annotations. For example, the software tool ANNOVAR, that functionally annotates genetic variants, is known to generate misclassifications. In contrast, the Ensembl Variant Effect Predictor (VEP) for example is a more accurate tool showing a 99,6% concordance with HGVS nomenclature in the study of Tuteja et al.^26^.

To facilitate inter-patient comparisons, as part of real-world data analyses, or intra-patient comparisons, (when performing a re-biopsy), it is essential to follow a consistent naming convention for *EGFR* exon20ins. In addition, as different *EGFR* exon20ins may result in different conformations on the protein level, not every mutation will respond to targeted therapy in the same way^6,18,19^. It is therefore of utmost importance to differentiate distinct *EGFR* exon20ins variants.

Non-compliance with HGVS recommendations when reporting biomarker variants is a known problem of external quality assessments^27^. While compliance has increased over the years, especially upon repeat participation of laboratories in multiple proficiency tests, there is still room for improvement with regards to clinical test reporting^27^. Our results further support this finding.

Inconsistent nomenclature of somatic variants also concerns descriptions of *EGFR* exon20ins in datasheets accompanying certain mutation-/allele-specific (q)PCR kits. In 6 different (q)PCR manuals, the mutation p.His773dup is described in three different ways (p.H773_V774insH, c.2319_2320insCAC and p.His773_V774insH; Fig. S2) and is only annotated correctly according to HGVS in one datasheet.

In summary, these two independent international proficiency tests have demonstrated that in large parts of Europe, a broad range of *EGFR* exon20ins can be detected reliably in routine tissue and liquid biopsy samples, if the test is suitable and validated. In terms of sample suitability, both, the internal and external proficiency tests have shown that—if processed correctly—artificial samples are a good alternative to routine pathology samples.

## Materials and methods

### Compilation of the mutational spectrum and clinical samples

To collate the mutational spectrum for the internal as well as the external proficiency tests, an extensive retrospective database analysis of clinical samples which had been examined by targeted NGS at the Institute of Pathology at the University of Cologne, Faculty of Medicine and University Hospital Cologne between 2013 and 2021 was performed, and the prevalence of the different *EGFR* exon20ins were calculated (study was approved by the Medical Ethics Committee of the University of Cologne, Faculty of Medicine, and University Hospital Cologne [ethical approval no. 22-1343-retro]). Clinical samples suitable for the proficiency tests were identified; samples were collected with informed consent from each patient and under approval of local ethical protocols. All methods were performed in accordance with the relevant guidelines and regulations of the Deutsche Akkreditierungsstelle (DAkkS). All patients´ samples were resection specimens of the lung. Tumor type was determined as non-small cell lung cancer (NSCLC). Tumor cell content of the samples was determined by experienced, board certified pathologists and had to be at least 30%. Necrosis could not exceed 10%. Furthermore, samples with one of the most common *EGFR* ex20ins mutation were chosen. As for clinical samples, two different mutations were chosen (p.Ala767_Val769dup and p.Ser768_Asp770dup) showing at least an allelic fraction of more than 20% and a coverage of > 200 × in the NGS analysis to be reliably detectable.

### Internal proficiency test

To determine the suitability of pre-selected reference samples for external *EGFR* exon20ins proficiency testing, an internal *EGFR* exon20ins proficiency test was established by the QuIP® lead panel institute, the Institute of Pathology at the University of Cologne, Faculty of Medicine and University Hospital Cologne.

#### Proficiency test specimens

Tissue reference samples comprised either pseudo-anonymized clinical or artificial FFPE material. Artificial samples were specifically provided by SensID GmbH (Rostock, Germany): the clinically relevant mutation sequences were synthesized as high fidelity dsDNA fragments with a size distribution similar to cfDNA/ctDNA by an EN ISO 13485-certified service provider. For artificial FFPE samples the mutation sequences were used to change the genomic profile of BDXXP4 cells (lymphoblastoid wt cell line), which were subsequently converted to FFPE cell blocks and sectioned with a microtome. Cell density was adjusted to yield > 400 ng DNA/section as determined by fluorometry (Qubit, Thermo Fisher Scientific Inc., Waltham, MA, USA).

Functionality and allelic fraction of artificial FFPE samples were tested by validated ddPCR assays (Bio-Rad QX200, Bio-Rad Laboratories, Hercules, California, USA). Each batch of artificial samples was quality-controlled for DNA quantity and VAF according to ISO 2850 (Acceptable Quality Levels) prior to testing for compatibility with NGS methods by lead panel institutes. All manufacturing steps and assays followed EN ISO 13485-certified standard operating procedures.

Cross-validation of the tissue samples for *EGFR* exon20ins was performed by six panel institutes based in Germany: the Institute of Tissue Diagnostics, MVZ at Helios Klinikum Emil von Behring, Berlin, the Institute of Pathology at the University Hospital Dresden, the Institute of Pathology at the University Hospital Erlangen, the Institute of Surgical Pathology at the Medical Center Freiburg, the Institute of Pathology at the University Hospital Halle (Saale) and the Institute of Pathology at the University Hospital Regensburg.

Panel institutes each received four sections of FFPE tissue mounted on glass slides: one 2 µm and two 10 µm-thick sections (clinical samples), or one 10 µm thick section in a screw cap tube (artificial samples).

For artificial liquid biopsy samples, gDNA was purified from Ashkenazim Son cells (GM24385, wt) and sheared ultrasonically to achieve a size profile of 167 ± 25 bp. Mutation sequences were spiked to achieve different variant allele frequencies (VAF) and added to the wt DNA. The DNA mix was added to DNA-free human plasma (SensID GmbH, Rostock, Germany) for a final concentration of 30 ng/ml. Functionality and allelic fraction determination were the same as for artificial FFPE samples.

Liquid biopsy reference samples were counter-tested for *EGFR* exon20ins by three of the six panel institutes: the Institute of Tissue Diagnostics, MVZ at Helios Klinikum Emil von Behring, Berlin, the Institute of Pathology at the University Hospital Dresden and the Institute of Clinical Pathology at the University Hospital Freiburg. Only artificial samples were used: panel institutes received 2 ml of human plasma spiked with 60 ng of fragmented wild-type or mutant DNA at various allelic fractions above 1%. To resemble patient material, fragment sizes ranged between 50 and 490 base pairs (bp), with a peak at 170 bp.

### External proficiency test

Following successful cross-validation of the tissue and liquid biopsy reference samples, two external *EGFR* exon20ins proficiency tests were launched: one covering institutes from Germany, Austria, and Switzerland only (further referred to as “DACH”) and one covering institutes from Austria, Belgium, Estonia, France, Germany, Greece, Hungary, Ireland, Italy, Lithuania, Netherlands, Poland, Portugal, Russia, Slovakia, Switzerland, and Turkey (further referred to as “international”). One institute from Germany, which had already participated in the DACH proficiency test, re-submitted results for the liquid biopsy part of the international proficiency test. German, Austrian, and Swiss institutes taking part in the international proficiency test did not overlap with institutes participating in the DACH proficiency test. The detection method was not pre-specified by QuIP®, i.e., participating institutes were free to choose their approach to *EGFR* exon20ins testing. The instructions for the proficiency tests stated only the range of expected mutations (‘insertions of known significance in the region between amino acids D761 to C775’) and the minimal allelic fraction to be detected (‘The minimum allele frequency here is 1%’). Based on this information, the participants were responsible to choose a suitable molecular method that covers the range of expected mutations and provides a sufficient limit of detection.

#### Proficiency test specimens

Samples for the DACH and international proficiency tests differed with regard to numbers and types of *EGFR* exon20ins covered. For the tissue part of the DACH proficiency test, participating institutes were sent two FFPE clinical samples carrying *EGFR* exon20ins as well as eight artificial FFPE samples—two wildtype samples and six samples with *EGFR* exon20ins (Table 2). Each of the two clinical samples were sub-divided into two 10 µm-thick sections for *EGFR* exon20ins testing and one 2 µm-thick section for H&E staining. For artificial tissue, participating institutes received one 10 µm-thick section per sample, without a corresponding section for H&E staining. FFPE blocks derived from clinical material were sectioned by the lead panel institute, the Institute of Pathology at University Hospital Cologne, FFPE blocks derived from artificial material were prepared by SensID.

**Table 2:** Composition and reference values for the external proficiency test samples (tissue parts; DACH proficiency test).

Tissue part					
Case	*EGFR* exon20ins	c.HGVS	p.HGVS	Chromosome location hg38	sample type
1	Yes	c.2311_2319dup	p.Asn771_His773dup	7: 55181320–55181328	Artificial material
2	Yes	c.2317_2319dup	p.His773dup	7: 55181326–55181328	
3	Yes	c.2314_2319dup	p.Pro772_His773dup	7: 55181323–55181328	
4	Yes	c.2315_2320dup	p.His773_Val774insAlaHis	7: 55181324–55181329	
5	Yes	c.2308_2309insGTT	p.Asp770delinsGlyTyr	7: 55181317–55181318	
6	Yes	c.2310_2311insGGT	p.Asp770_Asn771insGly	7: 55181319–55181320	
7	No	–	–	–	
8	No	–	–	–	
9	Yes	c.2300_2308dup	p.Ala767_Val769dup	7: 55181309–55181317	Clinical material
10	Yes	c.2303_2311dup	p.Ser768_Asp770dup	7: 55181312–55181320	

c: DNA level; p: protein level; DACH: Germany, Austria, Switzerland; dup: duplication; ins: insertion; EGFR: epidermal growth factor receptor; *EGFR* exon20ins: insertion mutation in exon 20 of the *EGFR* gene; HGVS: Human Genome Variation Society; NSCLC: non-small cell lung cancer. Ref-Seq ID: NM_005228.

Samples for the liquid biopsy part of the DACH proficiency test comprised two wildtype samples, six samples carrying the same *EGFR* exon20ins as the respective artificial samples in the tissue part, and two samples with *EGFR* exon20ins from the sample pool but with different allelic fractions (Table 3). Reference samples were produced by SensID; participating institutes received 2 ml of human plasma spiked with 60 ng of fragmented DNA at different allelic fractions above 1%.

**Table 3:** Composition and reference values for the external proficiency test samples (Liquid Biopsy part; DACH proficiency test).

Liquid biopsy part					
Case	*EGFR* exon20ins	c.HGVS	p.HGVS	Chromosome location hg38	Target VAF
1	Yes	c.2311_2319dup	p.Asn771_His773dup	7: 55181320–55181328	10
2	Yes	c.2311_2319dup	p.Asn771_His773dup	7: 55181320–55181328	1
3	Yes	c.2317_2319dup	p.His773dup	7: 55181326–55181328	10
4	Yes	c.2317_2319dup	p.His773dup	7: 55181326–55181328	2
5	Yes	c.2314_2319dup	p.Pro772_His773dup	7: 55181323–55181328	5
6	Yes	c.2315_2320dup	p.His773_Val774insAlaHis	7: 55181324–55181329	5
7	Yes	c.2308_2309insGTT	p.Asp770delinsGlyTyr	7: 55181317–55181318	5
8	Yes	c.2310_2311insGTT	p.Asp770_Asn771insGly	7: 55181319–55181320	5
9	No	–	–	–	0
10	No	–	–	–	0

VAF: variant allelic fraction; c: DNA level; p: protein level; DACH: Germany, Austria, Switzerland; dup: duplication; ins: insertion; EGFR: epidermal growth factor receptor; *EGFR* exon20ins: insertion mutation in exon 20 of the *EGFR* gene; HGVS: Human Genome Variation Society; NSCLC: non-small cell lung cancer. Ref-Seq ID: NM_005228.

For the tissue part of the international proficiency test, participating institutes received five artificial FFPE samples—one wildtype sample and four samples carrying different *EGFR* exon20ins—each with a thickness of 10 µm (Table 4). Test sets for the liquid biopsy part comprised one wildtype sample, two samples carrying the same *EGFR* exon20ins as the respective artificial samples in the tissue part, and two samples with *EGFR* exon20ins from the sample pool but with different allelic fractions (Table 4). As before, 2 ml of human plasma were spiked with 60 ng of fragmented DNA. All samples were prepared by SensID. The international proficiency test comprised a different number of samples compared to the national proficiency test due to the limited availability of clinical samples and the cost–benefit calculation for participating institutes.

**Table 4:** Composition and reference values for the external proficiency test samples (tissue and liquid biopsy parts; international proficiency test).

Tissue part					
Case	*EGFR* exon20ins	c.HGVS	p.HGVS	Chromosome location hg38	Sample type
1	Yes	c.2300_2308dup	p.Ala767_Val769dup	7:55181309–55181317	Artificial material
2	Yes	c.2303_2311dup	p.Ser768_Asp770dup	7:55181312–55181320	
3	No	–	–	–	
4	Yes	c.2315_2320dup	p.His773_Val774insAlaHis	7: 55181324–55181329	
5	Yes	c.2308_2309insGTT	p.Asp770delinsGlyTyr	7: 55181317–55181318	

Liquid biopsy part					
Case	*EGFR* exon20ins	c.HGVS	p.HGVS	Chromosome location hg38	Target VAF
1	Yes	c.2315_2320dup	p.His773_Val774insAlaHis	7: 55181324–55181329	5
2	Yes	c.2308_2309insGTT	p.Asp770delinsGlyTyr	7: 55181317–55181318	5
3	No	–	–	–	–
4	Yes	c.2311_2319dup	p.Asn771_His773dup	7: 55181320–55181328	10
5	Yes	c.2317_2319dup	p.His773dup	7: 55181326–55181328	2

VAF: variant allelic fraction; c: DNA level; p: protein level; dup: duplication; ins: insertion; EGFR: epidermal growth factor receptor; *EGFR* exon20ins: insertion mutation in exon 20 of the *EGFR* gene; HGVS: Human Genome Variation Society. Ref-Seq ID: NM_005228.

#### Proficiency test evaluation

For the DACH proficiency test, participating institutes were given 20 working days to perform their analyses and submit their results to the QuIP® website. Two points were awarded for each correctly identified sample (mutation detected ‘yes’ or ‘no’), resulting in a maximum score of 10 samples × 2 points = 20 points per part. If the sample could not be evaluated due to technical problems, one point was awarded per sample. The passing score for both parts was 90%, i.e., the total required score for successful participation was 18 points per part.

For the international proficiency test, participating institutes were initially given 20 working days to perform their analyses and submit their results to the QuIP® website. However, due to supply difficulties of several manufacturers reported by numerous institutes in various countries due to the corona virus pandemic, the submission deadline was later extended to 40 working days. Two points were awarded for each correctly identified sample (mutation detected ‘yes’ or ‘no’), resulting in a maximum score of 5 samples × 2 points = 10 points per part. For the international proficiency test only five samples had to be analyzed, as consequence, not one sample could be excluded from evaluation due to technical problems. Therefore, the passing score for both parts was 100% due to the low number of samples to be tested.

### Supplementary Information

Supplementary Information.

## Data Availability

The datasets generated and analyzed during the current study by next generation sequencing are available in the BIG SUB; Big submission portal repository [https://bigd.big.ac.cn/gsa-human/browse/HRA006093].
