# Supplementary material for: Multinational proficiency tests for EGFR exon 20 insertions reveal that the assay design matters
Source: Sci Rep. 2024 Jun 6;14:13069. doi: 10.1038/s41598-024-63821-2 (PMC11156884; doi:10.1038/s41598-024-63821-2)
Supplement: Supplementary file 1 — Supplementary Information. [file 41598_2024_63821_MOESM1_ESM.docx]

***Supplemental data to manuscript***

**Multinational proficiency tests for** ***EGFR* exon 20 insertions reveal that the assay design matters**

Table S1: Prevalence of EGFR exon20ins in a Cologne cohort

| **variant c.HGVS** | **variant p.HGVS** | **patients [n]** | **%** |
| --- | --- | --- | --- |
| c.2300_2308dup | p.Ala767_Val769dup | 36 | 17.7 |
| c.2302_2303insCGCTGGCCA | p.Ala767_Ser768insThrLeuAla | 1 | 0.5 |
| c.2303_2311dup | p.Ser768_Asp770dup | 35 | 17.2 |
| c.2302_2305delinsCCCT | p.Ser768_Val769delinsProLeu | 1 | 0.5 |
| c.2303_2305delinsTCG | p.Ser768_Val769delinsIleLeu | 7 | 3.4 |
| c.2306_2307insTTGCGT | p.Val769_Asp770insCysVal | 1 | 0.5 |
| c.2308_2309insGGGGGG | p.Val769_Asp770insGlyGly | 1 | 0.5 |
| c.2307_2308insTCACACGTG | p.Val769_Asp770insSerHisVal | 1 | 0.5 |
| c.2307_2308insTCCAGCGTG | p.Val769_Asp770insSerSerVal | 2 | 1.0 |
| c.2308_2309insGGGTTGTGG | p.Val769_Asp770insGlyValVal | 2 | 1.0 |
| c.2307_2315dup | p.Asp770_Pro772dup | 6 | 3.0 |
| c.2308delinsAACAACA | p.Asp770delinsAsnAsnAsn | 1 | 0.5 |
| c.2308_2309insGTT | p.Asp770delinsGlyTyr | 10 | 4.9 |
| c.2308_2323dup | p.Asp770_Val774dup | 3 | 1.5 |
| c.2310_2311insGGT | p.Asp770_Asn771insGly | 5 | 2.5 |
| c.2310_2311insTAC | p.Asp770_Asn771insTyr | 5 | 2.5 |
| c.2310_2311insGGGTTT | p.Asp770_Asn771insGlyPhe | 1 | 0.5 |
| c.2312_2313insGGGCCA | p.Asp770_Asn771insLysGly | 1 | 0.5 |
| c.2311_2313dup | p.Asn771dup | 2 | 1.0 |
| c.2311_2316dup | p.Asn771_Pro772dup | 1 | 0.5 |
| c.2311delinsCACC | p.Asn771delinsHisHis | 1 | 0.5 |
| c.2311_2312insCAC | p.Asn771delinsThrHis | 1 | 0.5 |
| c.2311delinsGGTT | p.Asn771delinsGlyTyr | 3 | 1.5 |
| c.2312_2313insACCCCACCCCCA | p.Asn771delinsLysProHisProHis | 1 | 0.5 |
| c.2313_2314insACG | p.Asn771_Pro772insTyr | 1 | 0.5 |
| c.2312_2314dup | p.Asn771_Pro772insHis | 2 | 1.0 |
| c.2311_2319 dup | p.Asn771_His773dup | 21 | 10.3 |
| c.2312delinsGGGG | p.Asn771delinsArgGly | 1 | 0.5 |
| c.2313delinsAGGT | p.Asn771delinsLysGly | 1 | 0.5 |
| c.2314_2316delinsACCCCT | p.Pro772delinsThrPro | 1 | 0.5 |
| c.2314_2319dup | p.Pro772_His773dup | 3 | 1.5 |
| c.2318_2319insACA | p.Pro772_His773insGln | 1 | 0.5 |
| c.2315_2316insAGGCAACCC | p.Pro772_His773insGlyAsnPro | 1 | 0.5 |
| c.2317_2318insCTCACCCTC | p.Pro772_His773insProHisPro | 1 | 0.5 |
| c.2317_2318insGGAACCCCC | p.Pro772_His773insArgAsnPro | 1 | 0.5 |
| c.2317_2318insGGCCAACCC | p.Pro772_His773insArgProThr | 1 | 0.5 |
| c.2316delinsAACCCAACCCACCCA | p.Pro772_His773insThrGlnProAsnPro | 1 | 0.5 |
| c.2317_2318insACAGGCAACCCC | p.Pro772_His773insHisArgGlnPro | 1 | 0.5 |
| c.2317_2319dup | p.His773dup | 7 | 3.4 |
| c.2316delinsTACAACCCCT | p.His773delinsTyrAsnProTyr | 1 | 0.5 |
| c.2317delinsAACCCCAACCCCT | p.His773delinsAsnProAsnProTyr | 1 | 0.5 |
| c.2315_2320dup | p.His773_Val774insAlaHis | 6 | 3.0 |
| c.2317_2322dup | p.His773_Val774dup | 10 | 4.9 |
| c.2318_2320delinsTCA | p.His773_Val774delinsLeuMet | 4 | 2.0 |
| c.2319_2320insCACCCCCAC | p.His773_Val774insHisProHis | 1 | 0.5 |
| c.2319_2320insCACAACCCCCAC | p.His773_Val774insHisAsnProHis | 1 | 0.5 |
| c.2319_2320insCCCCACCCCCAC | p.His773_Val774insProHisProHis | 2 | 1.0 |
| c.2320_2321insGCAACCCCCACG | p.His773_Val774insGlyAsnProHis | 1 | 0.5 |
| c.2320_2321insGCCACCCCCACG | p.His773_Val774insGlyHisProHis | 2 | 1.0 |
| c.2367_2378dup | p.Ile789_Leu792dup | 2 | 1.0 |

c: DNA level; p: protein level; *EGFR* exon20ins: insertion mutation in exon 20 of the *EGFR* gene; HGVS: Human Genome Variation Society. Ref-Seq ID: NM_005228.

Table S2: Results of the EGFR exon20ins analysis (tissue part; DACH proficiency test)

| **Result submitted** | **Case 1** | **Case 2** | **Case 3** | **Case 4** | **Case 5** | **Case 6** | **Case 7** | **Case 8** | **Case 9** | **Case 10** |
| --- | --- | --- | --- | --- | --- | --- | --- | --- | --- | --- |
| Yes (mutated) | *33* | *37* | *36* | *32* | *31* | *37* | 0 | 0 | *35* | *34* |
| No (not mutated) | 4 | 0 | 1 | 5 | 6 | 0 | *36* | *37* | 2 | 3 |
| Not evaluable | 0 | 0 | 0 | 0 | 0 | 0 | 1 | 0 | 0 | 0 |

Reference values are underlined and in italics.

DACH: Germany, Austria, Switzerland; EGFR: epidermal growth factor receptor; *EGFR* exon20ins: insertion mutation in exon 20 of the *EGFR* gene

Table S3: Detection kits used by participants for EGFR exon20ins testing (tissue part, DACH proficiency test)

| **Method** | **Manufacturer** | **Detection kit** | **No. of participants** | **With success (%)** |
| --- | --- | --- | --- | --- |
| Mutation/allele-specific (q)PCR | Biocartis | Idylla^TM^ EGFR Mutation Test | 3 | 0 (0) |
|  | Roche | cobas^®^ EGFR Mutation Test v2 | 1 | 0 (0) |
|  | AmoyDX | EGFR 29 Mutations Detection Kit | 1 | 0 (0) |
|  |  | Super-ARMS^®^ EGFR Mutation Detection Kit | 1 | 1 (100) |
|  | none | no specification | 1 | 1 (100) |
| Next-generation sequencing (parallel sequencing, NGS) | Agilent Technologies | SureSelect XT HS2 DNA Reagent Kit Custom Panel | 1 | 1 (100) |
|  | AmoyDx | HANDLE Classic NGS Panel | 1 | 1 (100) |
|  | Diagnostica Longwood | Archer^®^ VariantPlex^®^ Comprehensive Thyroid & Lung (CTL) Kit | 2 | 2 (100) |
|  | Illumina | AmpliSeq for Illumina Cancer Hotspot Panel v2 | 2 | 2 (100) |
|  | New Oncology | NEOselect v1 | 1 | 1 (100) |
|  | Qiagen | QIAseq Targeted DNA Custom Panel | 6 | 6 (100) |
|  | Thermo Fisher Scientific | Ion AmpliSeq™ Cancer Hotspot Panel v2 | 2 | 2 (100) |
|  |  | Ion AmpliSeq™ Colon and Lung Cancer Research Panel v2 | 1 | 1 (100) |
|  |  | Oncomine™ Focus Assay | 1 | 1 (100) |
|  |  | Oncomine™ Precision Assay | 2 | 2 (100) |
|  | Illumina, Thermo Fisher Scientific, Qiagen | nNGM Panel v2.0 | 4 | 4 (100) |
|  | none | Laboratory-developed test | 1 | 1 (100) |
| Pyrosequencing | Promega | PyroMark enzymes + customized primers | 1 | 1 (100) |
| Sanger sequencing | none | Laboratory-developed test + customized primers | 3 | 3 (100) |
|  | Thermo Fisher Scientific | BigDye™ Terminator Version 3.1 | 1 | 1 (100) |
|  | none | no specification | 1 | 1 (100) |

DACH: Germany, Austria, Switzerland; EGFR: epidermal growth factor receptor; *EGFR* exon20ins: insertion mutation in exon 20 of the *EGFR* gene; q: quantitative

Table S4: Results of the EGFR exon20ins analysis (liquid biopsy part; DACH proficiency test)

| **Result submitted** | **Case 1** | **Case 2** | **Case 3** | **Case 4** | **Case 5** | **Case 6** | **Case 7** | **Case 8** | **Case 9** | **Case 10** |
| --- | --- | --- | --- | --- | --- | --- | --- | --- | --- | --- |
| Yes (mutated) | *15* | *9* | *18* | *14* | *13* | *12* | *14* | *17* | 1 | 1 |
| No (not mutated) | 3 | 8 | 0 | 3 | 5 | 6 | 4 | 1 | *17* | *17* |
| Not evaluable | 0 | 0 | 0 | 1 | 0 | 0 | 0 | 0 | 0 | 0 |

Reference values are underlined and in italics.

DACH: Germany, Austria, Switzerland; EGFR: epidermal growth factor receptor; *EGFR* exon20ins: insertion mutation in exon 20 of the *EGFR* gene

Table S5: Detection kits used by participants for EGFR exon20ins testing (liquid biopsy part; DACH proficiency test)

| **Method** | **Manufacturer** | **Detection kit** | **No. of participants** | **With success (%)** |
| --- | --- | --- | --- | --- |
| Mutation/allele-specific (q)PCR | Biocartis | Idylla^TM^ EGFR Mutation Test | 2 | 0 (0) |
|  | Roche | cobas^®^ EGFR Mutation Test v2 | 1 | 0 (0) |
|  | AmoyDX | Super-ARMS^®^ EGFR Mutation Detection Kit | 1 | 0 (0) |
| Next-generation sequencing (parallel sequencing, NGS) | Diagnostica Longwood | Archer^®^ VariantPlex^®^ Comprehensive Thyroid & Lung (CTL) Kit | 1 | 1 (100) |
|  | Qiagen | QIASeq nNGM Panel v2.0 | 1 | 1 (100) |
|  |  | QIAseq Targeted DNA Custom Panel | 3 | 3 (100) |
|  | Thermo Fisher Scientific | Ion AmpliSeq™ Cancer Hotspot Panel v2 | 1 | 1 (100) |
|  |  | Ion AmpliSeq™ Colon and Lung Cancer Research Panel v2 | 1 | 1 (100) |
|  |  | Oncomine™ Lung cfDNA Assay | 5 | 3 (60) |
|  | none | Laboratory-developed test | 1 | 1 (100) |
| Fragment analysis | none | Laboratory-developed test | 1 | 1 (100) |

DACH: Germany, Austria, Switzerland; EGFR: epidermal growth factor receptor; *EGFR* exon20ins: insertion mutation in exon 20 of the *EGFR* gene; q: quantitative

Table S6: Results of the EGFR exon20ins analysis (tissue and liquid biopsy parts; international proficiency test)

| **Result submitted** | **Tissue part** | **Case 1** | **Case 2** | **Case 3** | **Case 4** | **Case 5** | **Liquid biopsy part** | **Case 1** | **Case 2** | **Case 3** | **Case 4** | **Case 5** |
| --- | --- | --- | --- | --- | --- | --- | --- | --- | --- | --- | --- | --- |
| Yes (mutated) |  | *62* | *65* | 4 | *55* | *48* |  | *25* | *27* | 0 | *35* | *42* |
| No (not mutated) |  | 5 | 2 | *63* | 12 | 19 |  | 23 | 21 | *48* | 13 | 6 |
| Not evaluable |  | 0 | 0 | 0 | 0 | 0 |  | 0 | 0 | 0 | 0 | 0 |

Reference values are underlined and in italics.

EGFR: epidermal growth factor receptor; *EGFR* exon20ins: insertion mutation in exon 20 of the *EGFR* gene

Table S7: Detection kits used by participants for EGFR exon20ins testing (tissue part; international proficiency test)

| **Method** | **Manufacturer** | **Detection kit** | **No. of participants** | **With success (%)** |
| --- | --- | --- | --- | --- |
| Mutation/allele-specific (q)PCR | Biocartis | Idylla^TM^ EGFR Mutation Test | 7 | 0 (0) |
|  | Diatech | EasyPGX ready EGFR | 3 | 0 (0) |
|  | AmoyDX | Super-ARMS^®^ EGFR Mutation Detection Kit | 1 | 1 (100) |
|  |  | AmoyDX EGFR 29 Mutations Detection Kit | 2 | 0 (0) |
|  | Entrogen | EGFR Mutation Analysis Kit for Real-Time PCR | 3 | 0 (0) |
|  | Roche | cobas^®^ EGFR Mutation Test v2 | 2 | 1 (50) |
| Next-generation sequencing (parallel sequencing, NGS) | Agilent | SureSelect XTHS custom panel | 1 | 1 (100) |
|  | Roche | AVENIO ctDNA Targeted Kit | 1 | 1 (100) |
|  | Vela Dx | Sentosa SQ OncoKey Focus | 2 | 1 (50) |
|  | Sophia Genetics | Sophia Genetics Solid Tumor Solution | 2 | 1 (50) |
|  | Diagnostica Longwood | ArcherDX^®^ Comprehensive Thyroid and Lung (CTL) Kit | 3 | 3 (100) |
|  | Illumina | AmpliSeq FOCUS DNA | 2 | 1 (50) |
|  |  | TruSight™ Tumor 170 | 1 | 1 (100) |
|  | Qiagen | GeneRead™ QIAact Lung DNA UMI Panel | 1 | 0 (0) |
|  |  | QIAseq Targeted DNA Custom Panel | 5 | 5 (100) |
|  | Thermo Fisher Scientific | Oncomine™ Lung cfDNA Assay | 1 | 0 (0) |
|  |  | Oncomine™ Solid Tumor | 2 | 2 (100) |
|  |  | Oncomine™ Dx Target Test | 1 | 1 (100) |
|  |  | Oncomine™ Precision Assay | 2 | 2 (100) |
|  |  | Oncomine™ Focus Assay | 5 | 5 (100) |
|  |  | Oncomine™ Comprehensive Assay | 2 | 2 (100) |
|  |  | Ampliseq™ Customized Panel | 4 | 4 (100) |
|  |  | Ion AmpliSeq™ Cancer Hotspot Panel v2 | 2 | 2 (100) |
|  |  | Ion AmpliSeq™ Colon and Lung Cancer Research Panel v2 | 4 | 3 (75) |
|  |  | Custom panel | 2 | 2 (100) |
|  | none | Laboratory-developed test | 4 | 4 (100) |
| Sanger sequencing | none | Laboratory-developed test | 1 | 1 (100) |
|  | Thermo Fisher Scientific | AppliedBiosystems™ BigDye Terminator V3.1 Cycle Sequencing Kit | 1 | 1 (100) |

EGFR: epidermal growth factor receptor; *EGFR* exon20ins: insertion mutation in exon 20 of the *EGFR* gene; q: quantitative

Table S8: Detection kits used by participants for EGFR exon20ins testing (liquid biopsy part; international proficiency test)

| **Method** | **Manufacturer** | **Detection kit** | **No. of participants** | **With success (%)** |
| --- | --- | --- | --- | --- |
| Mutation/allele-specific (q)PCR | Roche | cobas^®^ EGFR Mutation Test v2 | 9 | 0 (0) |
|  | Biocartis | Idylla™ ctEGFR Mutation Assay | 2 | 0 (0) |
|  | Diatech | Easy PGX^®^ | 3 | 0 (0) |
|  | AmoyDx | Super-ARMS^®^ EGFR Mutation Detection Kit | 6 | 0 (0) |
| Next-generation sequencing (parallel sequencing, NGS) | Thermo Fisher Scientific | Ion AmpliSeq™ Colon and Lung Cancer Panel v2 | 3 | 2 (66.7) |
|  |  | Oncomine™ Solid Tumor | 1 | 1 (100) |
|  |  | Oncomine™ Lung cfDNA Assay | 7 | 5 (71.4) |
|  |  | Oncomine™ Pan-Cancer Cell-Free Assay | 1 | 0 (0) |
|  | Illumina | AmpliSeq™ Cancer HotSpot Panel | 1 | 1 (100) |
|  |  | Ampliseq™ Design Custom Panel | 1 | 0 (0) |
|  |  | TruSight™ Tumor 170 | 1 | 1 (100) |
|  | Diagnostica Longwood | LIQUIDPlex ctDNA 28 | 2 | 2 (100) |
|  | Fluidigm | Custom panel | 1 | 1 (100) |
|  | Qiagen | GeneRead QIAact Actionable Insights Tumor Panel | 1 | 1 (100) |
|  |  | Gene Read UMI Advanced sequencing Q Kit | 1 | 1 (100) |
|  |  | Custom panel | 1 | 1 (100) |
|  | Sophia Genetics | Solid Tumor Solution | 2 | 2 (100) |
|  | Agilent | Magnis SureSelect XT HS Reagents | 2 | 2 (100) |
|  | none | Custom panel | 1 | 1 (100) |
| Pyrosequencing | none | none | 1 | 0 (0) |

EGFR: epidermal growth factor receptor; *EGFR* exon20ins: insertion mutation in exon 20 of the *EGFR* gene; q: quantitative

**Figure Legends**

**
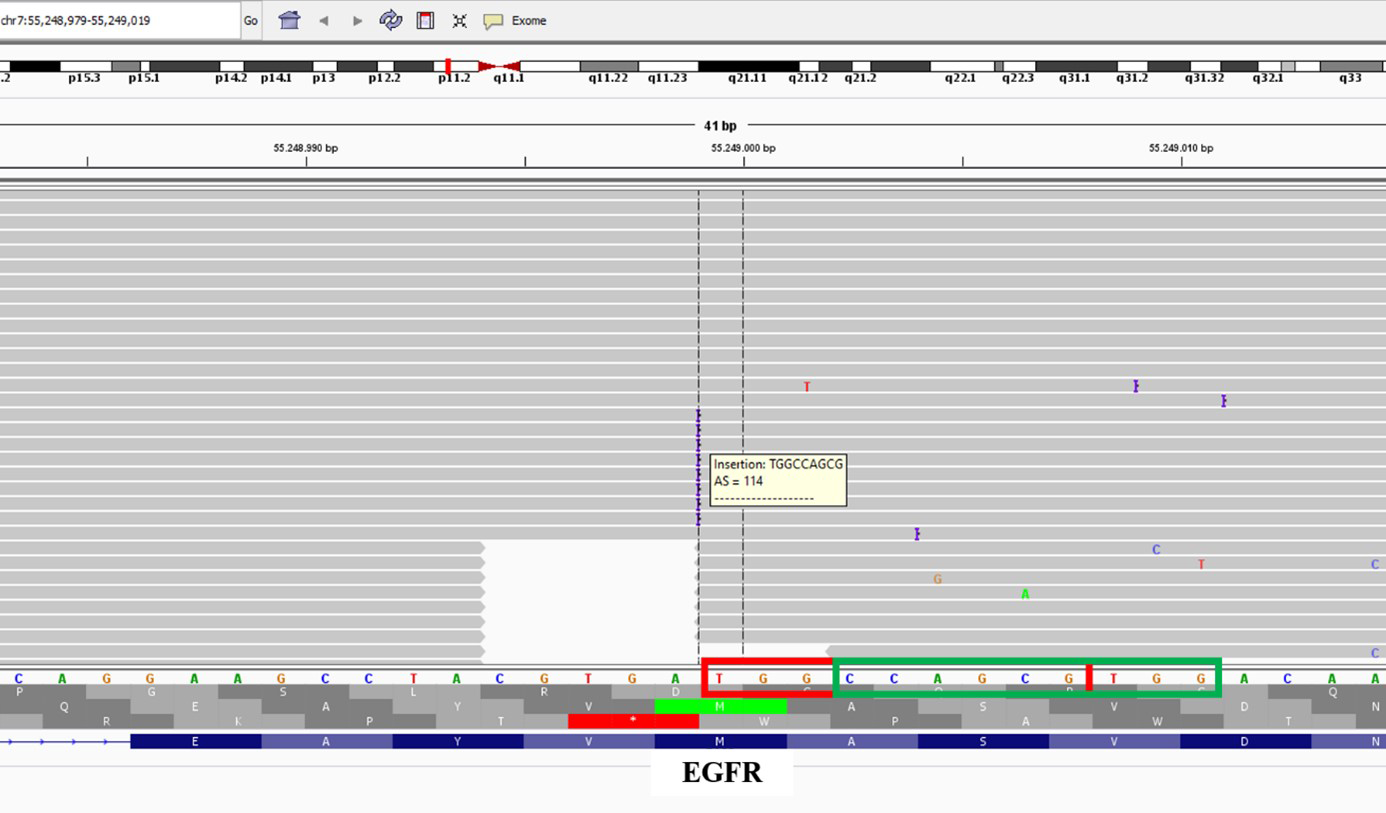
**

Figure S1: Example of correct nomenclature of p.Ala767_Val769dup (c.2300_2308dup) according to HGVS recommendations: duplications are to be prioritized over insertions and mutations are to be shifted to the most possible 3’ position of the reference sequence.

In the sample shown (sample 9 of the DACH proficiency test tissue part), nucleotides 2300 to 2308 are duplicated, resulting in a duplication of amino acids 767 to 769. The Human Genome Variation Society (HGVS) guidelines on the consistent and unambiguous description of sequence variants state that a duplication is to be prioritized when a variant can be described as either a duplication or an insertion. According to the **3’ rule**, which arbitrarily defines the most 3’ position possible of the reference sequence to have changed, the mutation description would need to be shifted towards the **3’** end on coding level (green rectangle) [1, 2]. Yet, it was often declared an insertion by participating institutes instead of a duplication and the **3’ rule was disregarded. This particular mutation was described on protein level as p.V769_D770insASV (or in three letter description: p.Val769_Asp770insAlaSerVal), p.S768_V769insVAS (or in three letter description: p.Ser768_Val769insValAlaSer) or as p.S768_D770dup (or in three letter description: p.Ser768_Asp770dup). On coding level, it was mostly described as insertion: c.2306_2307ins TGCCAGCGT, c.2306_2307ins9 or c.2307_2308insTGCCAGCGT.**


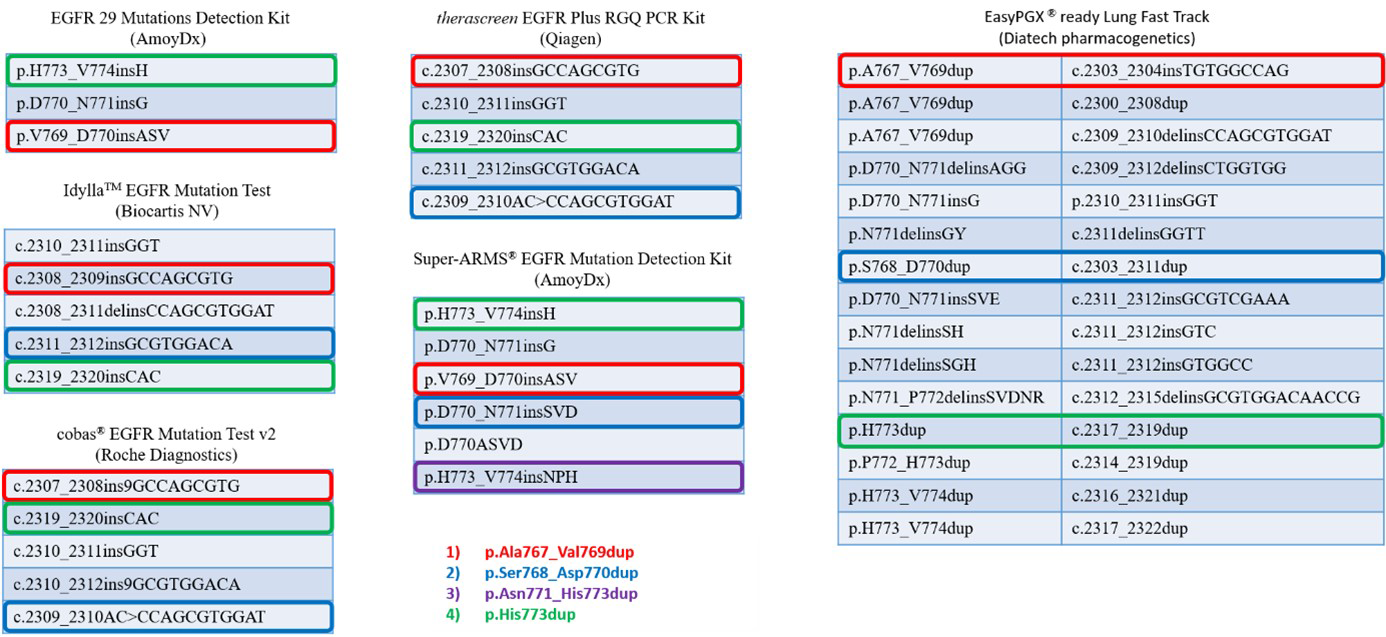


Figure S2: Comparison of six mutation-/allele-specific (q)PCR kits detecting EGFR exon20ins

Commercially available mutation-/allele-specific (q)PCR kits can only detect a limited number of *EGFR* exon20ins variants; the Idylla^TM^ EGFR Mutation Test and the cobas^®^ EGFR Mutation test v2, for example, only cover five distinct *EGFR* exon20ins each. As a reference: in a patient cohort from Cologne alone, 50 different *EGFR* exon20ins variants have been identified, some of them extremely rare.


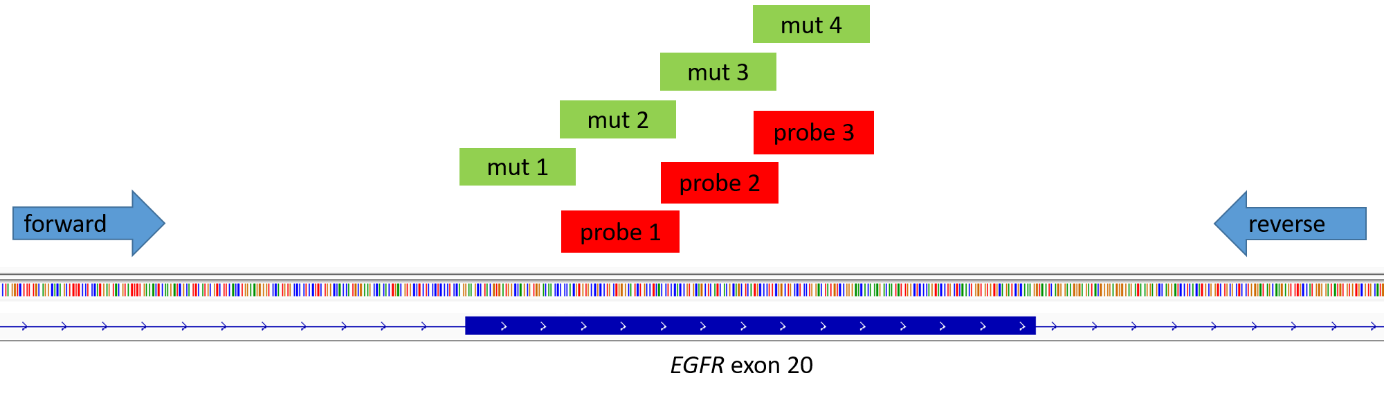


Figure S3: schematic presentation of the qPCR

The specificity of qPCR is gained by forward and reverse primers and probes that specifically bind to the target DNA. For each mutation a specific probe is needed. In case of *EGFR* ex20ins mutation detection, several probes have to be designed and at best multiplexed to cover the heterogeneity of the *EGFR* ex20ins mutations.

If a mutation (mut 1) is not covered by a specific probe, the qPCR fails to detect this mutation resulting in false-negative reports. Most commercially available qPCR assays cover only some of the many different *EGFR* ex20ins mutations, therefore mutations cannot be detected reliably.
